# Cardiovocal Syndrome (Ortner's Syndrome) Associated with Chronic Thromboembolic Pulmonary Hypertension and Giant Pulmonary Artery Aneurysm: Case Report and Review of the Literature

**DOI:** 10.1155/2012/230736

**Published:** 2012-10-14

**Authors:** Jaakko Heikkinen, Katrin Milger, Enrique Alejandre-Lafont, Christian Woitzik, Detlef Litzlbauer, Julia-Franziska Vogt, Jens Peter Klußmann, Ardeschir Ghofrani, Gabriele A. Krombach, Henning Tiede

**Affiliations:** ^1^Department of Radiology, Turku University Hospital, Kiinamyllynkatu 4-8; PL 52, 20521 Turku, Finland; ^2^Department of Radiology, University Hospital Giessen and Marburg, Justus Liebig University, Klinikstrasse 33, 35392 Gießen, Germany; ^3^Department of Internal Medicine, University of Giessen Lung Center, University Hospital Giessen and Marburg, Klinikstrasse 33, 35392 Gießen, Germany; ^4^Department of Otorhinolaryngology, Head and Neck Surgery, University Hospital Giessen and Marburg, Justus Liebig University, Klinikstrasse 33, 35392 Gießen, Germany

## Abstract

Cardiovocal syndrome or Ortner's syndrome is hoarseness due to left recurrent laryngeal nerve palsy caused by mechanical affection of the nerve from enlarged cardiovascular structures. Chronic thromboembolic pulmonary hypertension is extremely rarely found to cause this syndrome. We describe a case of a 56-year-old patient with sudden onset of hoarseness. The patient had known long standing severe pulmonary hypertension. Fiberoptic laryngoscopy showed left vocal cord palsy. Computed tomography of the neck and chest revealed extensive enlargement of the pulmonary arteries and excluded a malignant tumor. The diagnosis of cardiovocal syndrome was retained. It is important for the radiologist to be aware of this possible etiology causing left recurrent laryngeal nerve palsy and to understand its mechanism.

## 1. Introduction

Hoarseness of voice is a very common condition and underlying causes vary from reversible benign causes to life-threatening malignancies. Unilateral recurrent laryngeal nerve injury is most commonly caused by surgical trauma or a malignant tumor [1]. Cardiovocal syndrome or Ortner's syndrome is hoarseness due to left recurrent laryngeal nerve palsy caused mainly by mechanical affection of the nerve from enlarged cardiovascular structures. Cardiovocal syndrome is a rare condition and to our knowledge only 40 patients have been presented in the literature as a thorough research in PubMed showed. It is also described to be more common in men because possibly of a higher incidence of cardiovascular conditions than in females [2]. Cardiovocal syndrome has a peak incidence in older age, but can occur in any age group, and it has been described even in infants [3]. We present a case report of Ortner's syndrome of uncommon etiology that illustrates the underlying pathoanatomical mechanisms. 

## 2. Case Report

**Figure 1:**
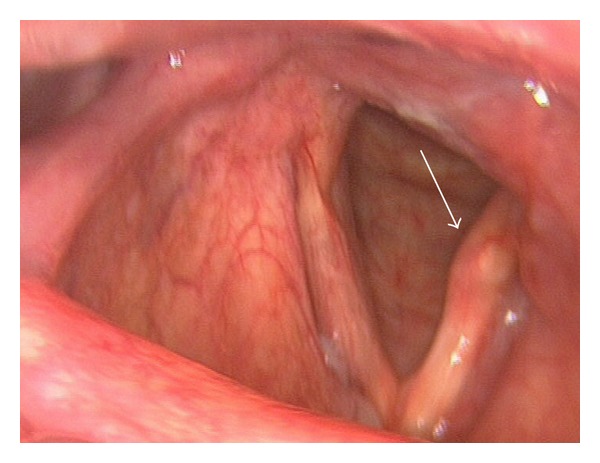
56-year-old male with chronic thromboembolic pulmonary hypertension and sudden hoarseness. Appearance of the paralysed left vocal cord (white arrow) during fiberoptic laryngoscopy, no malignant lesion was detected in this examination.

**Figure 2:**
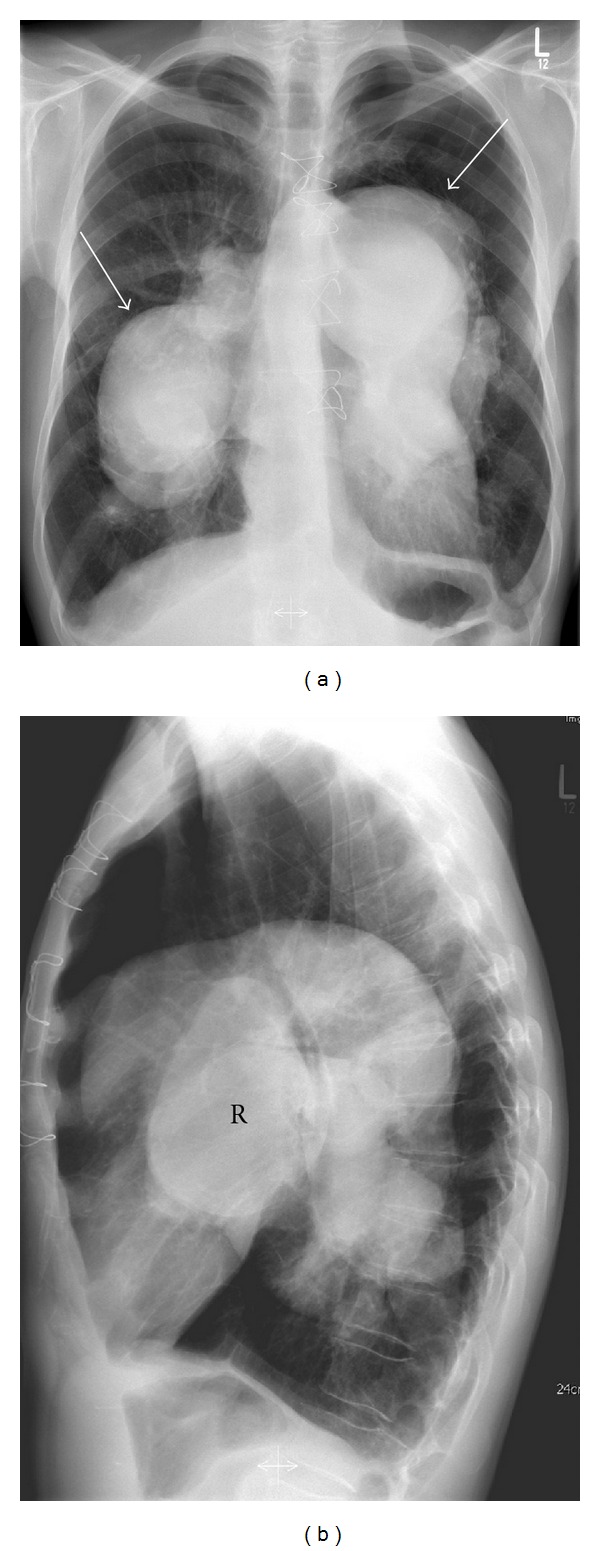
Chest radiographs ((a) and (b)) from 2012 showed severe dilatation of both pulmonary arteries (arrows). (R = right pulmonary artery).

**Figure 3:**
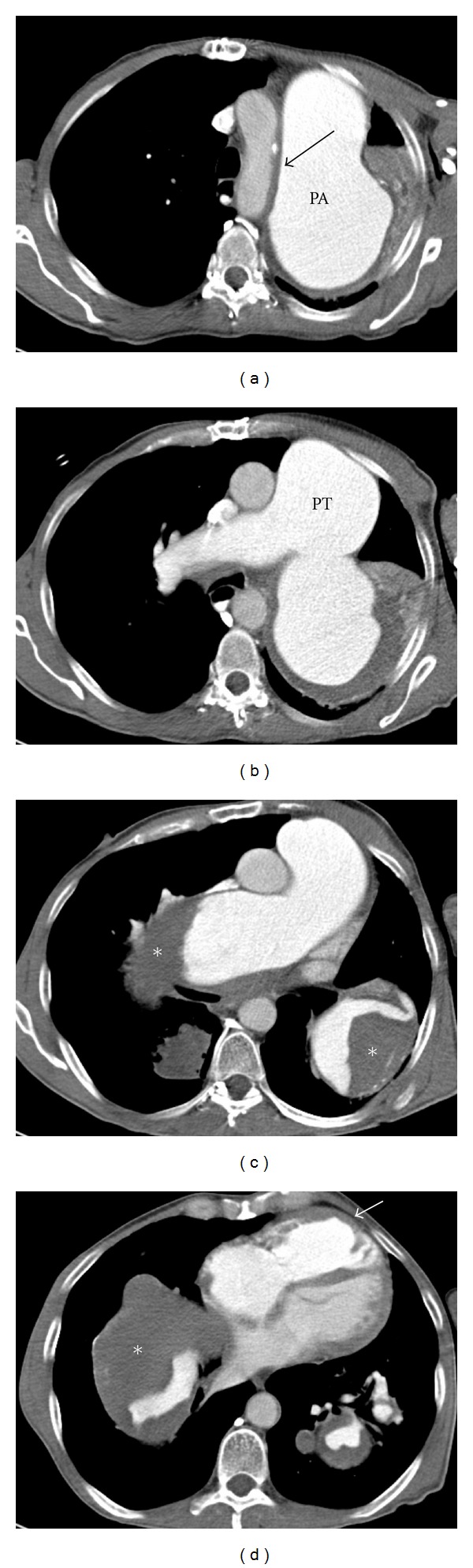
Chest CT featured the signs of pulmonary arterial hypertension, pulmonary trunk (PT) dilatation, aneurysmal dilatation of the central pulmonary arteries (PA), wall-adherent thrombotic material in pulmonary arteries (asterix), right ventricular hypertrophy (white arrow in (d)). No mass lesion or lymphadenopathy was found and the radiologist suggested the cardiovocal syndrome as a possible diagnosis because of the obvious mass effect caused by the dilated central pulmonary arteries. The possible compression of the left recurrent laryngeal nerve between the aorta and pulmonary artery is well appreciated (black arrow in (a)).

**Figure 4:**
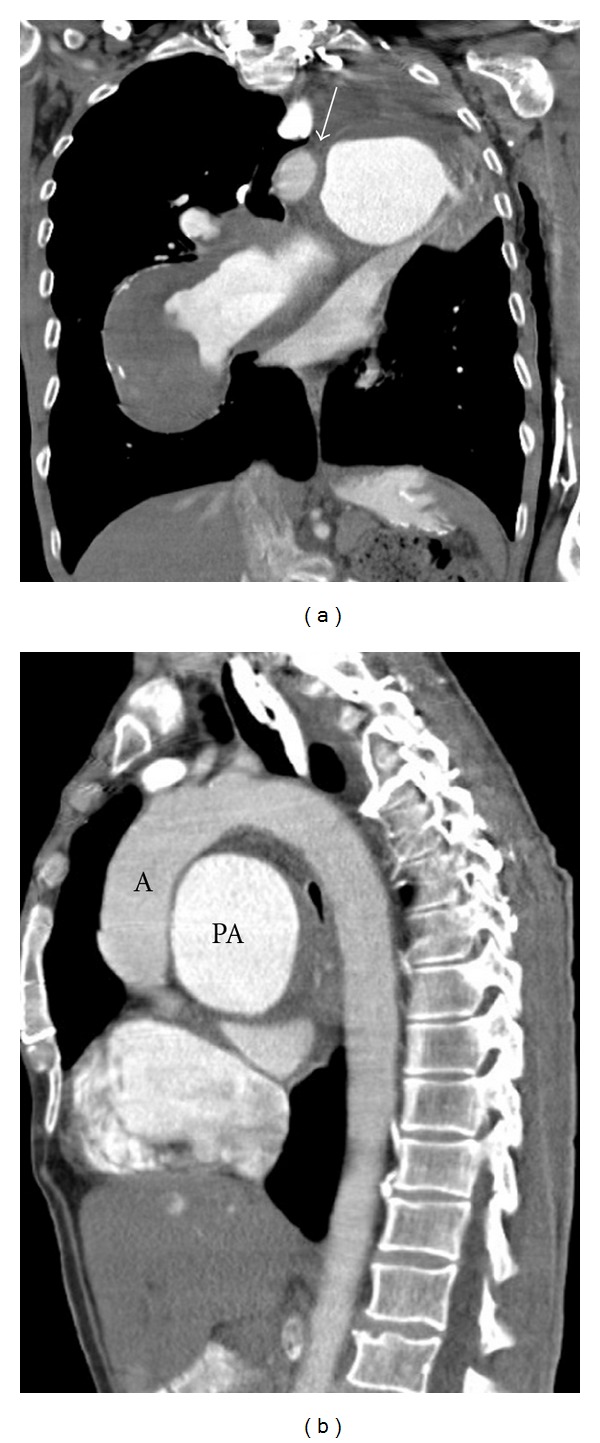
Coronal (a) and sagittal reformat (b) from the CT. The aortopulmonary window (arrow in (a)) is almost completely obstructed by the enlarged left pulmonary artery. (b) The sagittal reformat demonstrates the narrow space between the aorta (A) and pulmonary artery (PA).

A 56-year-old patient with a fifteen-year history of known severe chronic thromboembolic pulmonary hypertension (CTEPH) and chronic obstructive pulmonary disease (COPD) developed sudden hoarseness of voice. He had a history of smoking (over 25 pack years). Fiberoptic laryngoscopy revealed left vocal cord palsy (Figure 1), while the rest of the otolaryngologic exam was normal. Chest radiograph was relatively unchanged from previous studies obtained years ago and demonstrated massive dilatation of the pulmonary arteries, and tapering of peripheral pulmonary vessels (Figure 2). Contrast-enhanced computed tomography (CT) of the neck and chest was performed to evaluate the patient for a possible malignant process causing the symptoms. The CT examination did not show a tumor. Chest CT showed the classical signs of pulmonary arterial hypertension, including extensive central pulmonary artery dilatation, wall-adherent thrombus in the pulmonary arteries, abrupt narrowing, or tapering of peripheral pulmonary vessels, right ventricular hypertrophy, right ventricular and atrial enlargement, dilated bronchial arteries, and a mosaic pattern of attenuation due to variable lung perfusion (Figure 3). The aneurysmal dilatation of the pulmonary arteries (Figures 3 and 4) is a known, but extremely rare finding in chronic thrombembolic pulmonary hypertension. The central pulmonary artery had enlarged from 5.2 cm in 2002 to 7 cm in 2012. The massive aneurysmal enlargements of the right and left pulmonary arteries had also progressed from 4.7 cm and 5.6 cm to 7 cm and 9 cm, respectively. As there was no other possible explanation for the left recurrent laryngeal nerve palsy, mechanical compression of the left laryngeal nerve between the aorta and left pulmonary artery was most likely the cause. The clinical symptoms in combination with imaging findings were consistent with cardiovocal syndrome. A specialist for thoracic surgery was consulted but operative decompression was not possible due to the severe enlargement of the pulmonary arteries. The patient was discharged after diagnosis with maximal medical treatment of the pulmonary hypertension without relieve of hoarseness. Three months later the patient died from rupture of the aneurysm.

## 3. Discussion

The recurrent laryngeal nerves provide ipsilateral motor innervation to the intrinsic laryngeal muscles for vocalisation. The recurrent laryngeal nerves branch from the vagus nerve (the tenth cranial nerve) at different levels for the left and right side. The recurrent laryngeal nerves descend first into the thorax before rising up between the trachea and esophagus to reach the neck. The right recurrent laryngeal nerve branches at the level of the right subclavian artery and hooks around this artery. The left recurrent laryngeal nerve is longer and it branches from the vagus nerve at the level of the tranverse aortic arch. It hooks under the arch of aorta, posterior to the ligamentum arteriosum before ascending towards the neck between the trachea and the esophagus. Unilateral damage to the recurrent laryngeal nerve usually causes hoarseness, as in our patient, while bilateral damage to the nerves causes usually more severe symptoms. Because of the anatomical course of the recurrent laryngeal nerves, the left side is more vulnerable to damage. In unilateral vocal cord palsy due to thoracic diseases, left-sided vocal cord paralysis was 1.75 times more frequent than right-sided paralysis [4]. Damage to the left recurrent laryngeal nerve can happen at any level of the nerves course and in some cases it can be compressed between the aorta and pulmonary artery because of a very narrow space between these vessels, as in our patient. In malignant causes compression and also nerve invasion can cause damage to the nerve. Many possible causes of unilateral vocal cord paralysis are known, including malignant or less often benign tumors, iatrogenic injury, inflammation, or postradiation fibrosis causing nerve palsy due to enchasement [5]. In a recently published series of 115 patients, in 70 patients an intrathoracic cause of vocal cord paralysis could be identified. Neoplasm accounted for 36%, iatrogenic injury for 34%, benign tumors for 17%, and inflammation for 10%, while a vascular cause (an aortic arch aneurysm) occurred in one patient [4].

Cardiovocal syndrome was originally described in 1897 by Nobert Ortner in three patients with severe mitral stenosis [6]. He explained that hoarseness was caused by compression of the left recurrent laryngeal nerve by the enlarged left atrium. Later it has been encountered with other mediastinal structures causing mass effect [7, 8] and in many cardiac conditions for example, congenital heart diseases, mitral valve disorders, ventricular and aortic aneurysms, atrial enlargement and in iatrogenic conditions [9]. Cardiovocal syndrome caused by idiopathic pulmonary artery hypertension and dilated pulmonary trunk has also been described in the literature [10–12]. To the best of our knowledge cardiovocal syndrome associated with pulmonary embolism is very rare condition and it has been described only twice [13, 14]. Pathophysiological mechanism of this syndrome is thought to be compression of the left recurrent laryngeal nerve between the aorta and dilated pulmonary artery [12], and this can be well appreciated from the axial CT images and coronal and sagittal reformats (Figures 3 and 4). Our patient has had CTEPH and extensively dilated pulmonary arteries for many years. CT findings showed the progressive dilatation of the pulmonary trunk and arteries and indicated compression of the left recurrent laryngeal in the vulnerable space between aorta and pulmonary artery. Sudden development of hoarseness due to vocal-cord palsy in patients with aneurysm has been recognized as a possible predictor of catastrophic rupture of the aneurysm, since it is a sign of possible enlargement of the aneurysm, and thus can be considered as a prognostic factor [12].

Chest radiograph is usually ordered as the first imaging study, since it is readily accessible and can give hints of the underlying condition and direct further studies. Chest radiographs can reveal a chest malignancy as the cause of the left vocal-cord palsy and detect cardiomegaly and mediastinal masses.

Neck and chest CT and/or MRI should be done for all patients with left recurrent nerve palsy, because of the many possible etiologies. The imaging should include the entire course of the left recurrent laryngeal nerve. Imaging from the base of skull to the upper abdomen is usually done to cover the lungs although distal end of the nerve is at level of the aortic arch. CT is useful for evaluating the mediastinum and especially the aortopulmonary region which can be difficult in plain radiographs. In a larger study of patients with left recurrent laryngeal nerve palsy aortopulmonary area was normal in 72% of the cases on chest radiographs but CT demonstrated a mass lesion [15]. CT- and MRI-imaging modalities can be used for making the diagnosis, to evaluate the extent and location of the pathology and guiding treatment options such as surgery or radiation therapy. An intracranial isolated laryngeal-motor-nerve lesion without any other signs or symptoms is unlikely and brain MRI or CT is not routinely recommended. For male patients esophagography or esophagoscopy is recommended when no primary malignancy is found on the CT scans because of the higher incidence of esophageal cancer in males [3]. Fusion imaging using PET/CT or PET/MRI are new sophisticated multimodality methods, and these imaging choices could be increasingly used in the future for achieving better sensitivity and specificity.

Early recognition of the cause of the left recurrent laryngeal nerve palsy is the most important part of the treatment, because reversibility of the nerve damage depends on the duration of injury. Radiologists can rule out possible pulmonary and mediastinal masses and as in our case, and based on the findings suggest the etiology directing the treatment of the patient. Prognosis of this syndrome depends on the underlying cardiovascular condition and correction of the underlying cardiac or vascular anomaly is important to a successful recovery. If the cardiovascular condition of a patient does not allow for causal treatment and the symptoms are severely disabling, voice therapy or even surgical tightening of the affected vocal cord might be considered.

Since there are many conditions that can cause hoarseness including cancer of vocal cord and unilateral vocal-cord palsy including metastatic pulmonary carcinoma radiological imaging is used for differential diagnostics and detecting the underlying cause of the symptom [15]. The nerves of the thorax cannot be directly delineated on CT images, but understanding the functional anatomy and clinical significance of these nerves is important for the correct analysis of thoracic images [16]. The left recurrent laryngeal nerve is usually supplied by the anterior bronchoesophageal artery, while the right is supplied by the inferior thyroideal artery. Damage to one of these arteries during surgery is a known cause of iatrogen unilateral vocal cord palsy [17]. However, occlusion of the supplying artery from compression by the enlarged pulmonary arteries is very unlikely, due to the course of the vessel.

Cardiovocal syndrome is a rare cause for left recurrent laryngeal nerve palsy. The cardiovascular cause of the hoarseness might be of much higher consequence for the patient as the hoarseness itself, as in the presented case. Radiological imaging modalities are important for differentiating this syndrome from other diseases such as mediastinal masses or pulmonary cancer.
